# Long non-coding RNA UBE2CP3 promotes tumor metastasis by inducing epithelial-mesenchymal transition in hepatocellular carcinoma

**DOI:** 10.18632/oncotarget.18524

**Published:** 2017-06-16

**Authors:** Shun-Wang Cao, Jin-Lan Huang, Jing Chen, Yan-Wei Hu, Xiu-Mei Hu, Ting-Yu Ren, Shi-Hao Zheng, Jin-Duan Lin, Jing Tang, Lei Zheng, Qian Wang

**Affiliations:** ^1^ Laboratory Medicine Center, Nanfang Hospital, Southern Medical University, Guangzhou, Guangdong, China; ^2^ Department of Clinical Laboratory, First Affiliated Hospital of Fujian Medical University, Fuzhou, Fujian, China; ^3^ Department of Clinical Laboratory Medicine Center, Shenzhen Hospital of Southern Medical University, Shenzhen, Guangdong, China; ^4^ Department of Neurosurgery, Fujian Provincial Hospital, Fuzhou, Fujian, China; ^5^ Guangdong Provincial Key Laboratory of Gastroenterology, Department of Gastroenterology, Nanfang Hospital, Southern Medical University, Guangzhou, Guangdong, China

**Keywords:** long non-coding RNA, UBE2CP3, HCC, metastasis, EMT

## Abstract

Hepatocellular carcinoma (HCC) is a highly aggressive, solid malignancy that has a poor prognosis. Long non-coding RNAs (lncRNAs) have been found to be dysregulated in various cancers, including HCC. However, the molecular mechanism involving lncRNAs in HCC remains largely unknown. In this study, lncRNAs differentially expressed between HCC and corresponding non-cancerous tissue were identified by microarray analysis. A specific differentially expressed lncRNA UBE2CP3 (ubiquitin conjugating enzyme E2 C pseudogene 3) was identified. LncRNA UBE2CP3 was frequently up-regulated in HCC samples as assessed by quantitative real-time polymerase chain reaction (qRT-PCR) and *in situ* hybridization (ISH) experiments. Clinical data showed that high levels of lncRNA UBE2CP3 were correlated with poor prognosis in HCC patients. Functional studies demonstrated that over-expression of lncRNA UBE2CP3 promoted cell invasion and migration *in vitro* and *in vivo*. Mechanistically, enhanced expression of lncRNA UBE2CP3 increased the expression of Snail1 and N-cadherin, but decreased the expression of E-cadherin, thus promoting the process of epithelial to mesenchymal transition (EMT) and finally inducing cell invasion and migration. Furthermore, serum levels of lncRNA UBE2CP3 were increased in HCC patients and decreased after surgery. Our results suggest that lncRNA UBE2CP3 promotes the metastasis of HCC and that serum lncRNA UBE2CP3 may be a new biomarker for the diagnosis of HCC.

## INTRODUCTION

Hepatocellular carcinoma (HCC) is a highly aggressive malignancy that is associated with a poor prognosis [1]. The chief reasons responsible for the poor prognosis are the high rate of tumor recurrence and distant metastasis after hepatic resection [2]. Thus, it is of great importance to characterize the pathogenic mechanisms of HCC in order to identify novel early biomarkers and therapeutic targets for HCC.

Long non-coding RNAs (lncRNAs) are defined as transcribed RNA molecules greater than 200 nt in length, which show no protein-coding capacity [3, 4]. LncRNAs were initially considered as simply transcriptional “noise” or cloning artifacts [5]. However, mounting evidence has linked dysregulations of lncRNAs to cancers. LncRNAs may have oncogenic or tumor suppressive roles in the regulation of multiple biological processes such as development, differentiation and carcinogenesis [6–8]. Furthermore, lncRNAs are reported to play regulatory roles in cancer recurrence and metastasis and contribute to tumorigenesis and tumor progression [9–11]. However, to date, only a limited number of lncRNAs have been studied in the onset and progression of HCC.

LncRNAs are relatively stably expressed in human body fluids and thus their levels may be used as clinical indicators [12–14]. Plasma AA174084 levels have been reported to be associated with invasion and lymphatic metastasis, and the postoperative levels were found to be decreased compared with preoperative levels [15]. Additionally, circulating lncRNAs CUDR, LSINCT-5 and PTENP1 have been reported to distinguish patients with gastric cancer from healthy controls [16]. To date, circulating lncRNAs are seldom studied in the serum of HCC patients. Plasma HULC and Linc00152 were up-regulated dramatically in HCC patients, indicating a role as significant predictors of tumor growth and metastasis of HCC [17]. Moreover, lncRNAs RP11-160H22.5, XLOC_014172 and LOC149086 were identified as the potential biomarkers for the tumorigenesis. XLOC_014172 and LOC149086 were considered to be the potential biomarkers for metastasis prediction in HCC[18]. Accordingly, circulating lncRNAs may be a promising tool for the early detection of HCC.

In this study, differences between the lncRNA expression profiles for HCC and tumor-adjacent non-tumor tissue were assessed by microarray analysis. We found that lncRNA UBE2CP3 was up-regulated in HCC. UBE2CP3 (NCBI gene ID:100129983) is an abbreviation of ubiquitin conjugating enzyme E2 C pseudogene 3. It is a pseudogene transcribed from chromosome 4q12 and a novel lncRNA yet to be investigated in cancer. We analyzed expression and localization of lncRNA UBE2CP3 by quantitative real-time polymerase chain reaction (qRT-PCR) and *in situ* hybridization (ISH) with patient samples from two HCC cohorts. Further investigation of the biological function of lncRNA UBE2CP3 were performed through gain- and loss-of-function studies, which demonstrated that lncRNA UBE2CP3 provoked cell invasion and migration. Additionally, we examined the serum levels of lncRNA UBE2CP3 in HCC patients, the results of which suggest that lncRNA UBE2CP3 may be a clinically useful diagnostic biomarker for HCC.

## RESULTS

### LncRNA UBE2CP3 is up-regulated in HCC tissue

**Figure 1:**
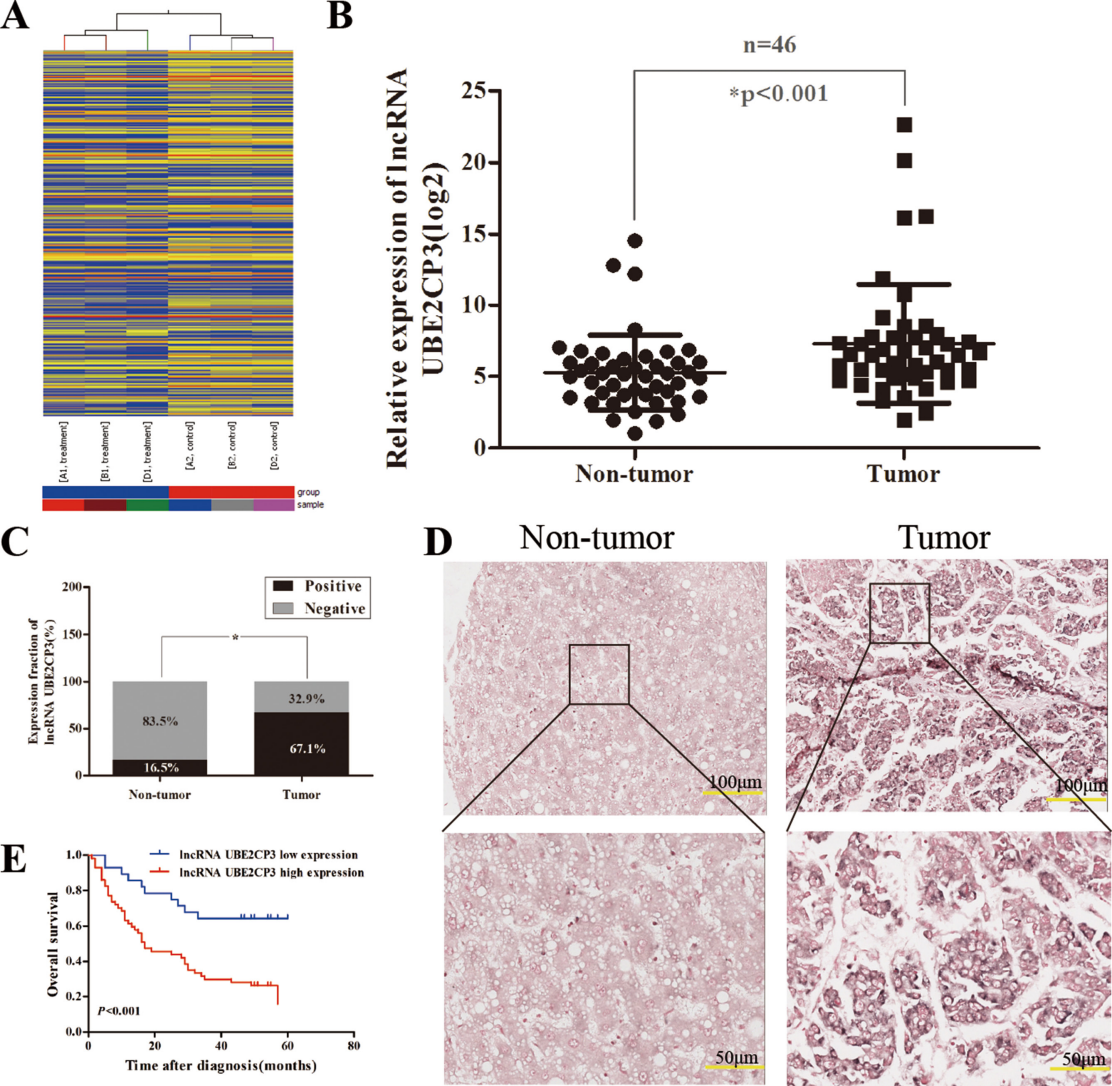
LncRNA UBE2CP3 is over-expressed in HCC tissue (**A**) Hierarchical clustering analysis of the differentially expressed lncRNAs (> 2-fold; *P* < 0.05) between HCC samples (A1, B1, D1, tumor) and paired non-tumor samples (A2, B2, D2, non-tumor). (**B**) LncRNA UBE2CP3 expression was detected by qRT-PCR in HCC samples and adjacent non-cancerous liver tissue (Cohort 1, *n* = 46). The level of lncRNA UBE2CP3 was normalized to that of U6. *P < 0.05. (**C**) LncRNA UBE2CP3 expression in HCC tissue and adjacent non-cancerous liver tissue was determined in ISH assays (Cohort 2, *n* = 85). *P < 0.05. (**D**) Representative images of lncRNA UBE2CP3 expression from tumor tissue and non-cancerous tissue by ISH assays. (**E**) Kaplan-Meier survival analysis of OS in HCC patients (*P* < 0.001) based on lncRNA UBE2CP3 expression. The differences were assessed by the log-rank test.

To identify lncRNAs that have the potential to drive liver tumorigenesis, a lncRNA expression profile was determined by microarray analysis. Hierarchical clustering showed systematic variations in transcript expression levels between paired tumor and non-tumor tissue from HCC patients (Figure 1A). The microarray data have been deposited in NCBI Gene Expression Omnibus and are accessible through GEO Series Accession Number GSE89186 (http://www.ncbi.nlm.nih.gov/geo/query/acc.cgi?acc=GSE89186). The microarray findings revealed a set of lncRNAs which were dysregulated in HCC tissue, of which lncRNA UBE2CP3 was one of the most up-regulated. To further validate the expression of lncRNA UBE2CP3 in HCC tissue, qRT-PCR was performed in 46 pairs of HCC/non-tumor tissue (Cohort 1). The lncRNA UBE2CP3 transcripts were expressed at higher levels in HCC tissue than non-tumor tissue from the same donor, after normalizing to U6 expression (*P* < 0.001; Figure 1B). The ISH studies performed with 85 paraffin-embedded HCC specimens from Cohort 2 confirmed the up-regulation of lncRNA UBE2CP3 in HCC and suggested that lncRNA UBE2CP3 was localized to the cytoplasm of HCC tissue (*P* < 0.001; Figure 1C and 1D).These findings indicate that lncRNA UBE2CP3 is frequently up-regulated in HCC.

### Association between lncRNA UBE2CP3 expression and clinicopathological characteristics

To determine whether the levels of lncRNA UBE2CP3 are related to HCC development, we analyzed the correlation between lncRNA UBE2CP3 and the clinicopathological characteristics in HCC patients of Cohort 1 and Cohort 2. Statistical analysis revealed that high levels of lncRNA UBE2CP3 were positively correlated with Edmondson grade (*P* = 0.017, Table 2) in Cohort 2. Moreover, Kaplan-Meier and log-rank test analysis suggested that HCC patients with high levels of lncRNA UBE2CP3 exhibited a reduced overall survival (OS) (*P* < 0.001; Figure 1E). However, no significant correlations were observed between lncRNA UBE2CP3 and other clinicopathological features, including gender, age, liver cirrhosis, tumor number and tumor size in Cohort 2. Nonetheless, in Cohort 1, we revealed that high levels of lncRNA UBE2CP3 were significantly correlated with Edmondson grade (*P* = 0.038, Table 1), smoking (*P* = 0.028, Table 1) and alcohol (*P* = 0.047, Table 1). In addition, univariate analysis of prognostic variables revealed that lncRNA UBE2CP3 expression (*P* = 0.001), Edmondson grade (*P* = 0.015), and tumor size (*P* = 0.012) were significantly related to overall survival in patients with HCC (Table 3). Multivariate analysis was performed using the Cox Proportional hazards model. The results showed that lncRNA UBE2CP3 expression (95%CI:1.333-5.540; *P* = 0.006) was an independent prognostic factor for HCC patients (Table 3).

**Table 1:** Correlation between lncRNA UBE2CP3 expression and HCC clinicopathologic features in 46 patients: Cohort 1

	lncRNA UBE2CP3 expression levels		^*^P
	Low expression	High expression#	
**All cases**	23	23	
**Age, y**			0.536
**< = 55**	16	14	
**> 55**	7	9	
**Gender**			0.381
**Male**	19	21	
**Female**	4	2	
**HBsAg**			0.753
**Positive**	16	15	
**Negative**	7	8	
**Tumor size, cm**			0.099
**< = 5**	13	5	
**> 5**	10	18	
**Edmondson grade**			0.038^*^
**I–II**	14	7	
**III–IV**	9	16	
**AFP level, ng/ml**			0.767
**< = 20**	13	12	
**> 20**	10	11	
**Cirrhosis**			0.546
**Yes**	13	15	
**No**	10	8	
**Tumors, n**			0.295
**Solitary**	22	20	
**Multiple**	1	3	
**Smoking**			0.028^*^
**Yes**	4	12	
**No**	19	11	
**Alcohol**			0.047^*^
**Yes**	4	15	
**No**	19	8	

^#^ The median expression level was used as the cutoff. Low expression of lncRNA UBE2CP3 in 22 patients was classified as values below the 50th percentile. High lncRNA UBE2CP3 expression in 23 patients was classified as values at or above the 50th percentile.

^*^For analysis of correlation between lncRNA UBE2CP3 levels and clinical features, Pearson’s chi-square tests were used. *P < 0 .05.

**Table 2:** Correlation between lncRNA UBE2CP3 expression and HCC clinicopathologic features in 85 patients: Cohort 2

	lncRNA UBE2CP3 expression levels		*P
	Low expression	High expression	
**Age, y**			0.823
**< = 55**	15	32	
**> 55**	13	25	
**Gender**			0.074
**Male**	22	53	
**Female**	6	4	
**Edmondson grade**			0.017^*^
**I + II**	27	43	
**III + IV**	1	14	
**Cirrhosis**			0.548
**With**	8	20	
**Without**	20	37	
**Tumors, n**			0.379
**Solitary**	27	52	
**Multiple**	1	5	
**Tumor size, cm**			0.099
**< = 5**	14	18	
**> 5**	14	39	
**BCLC stage**			0.561
**A**	9	22	
**B+C+D**	19	35	

^*^For analysis of correlation between lncRNA UBE2CP3 levels and clinical features, Pearson’s chi-square tests were used. *P < 0 .05.

**Table 3:** Univariate and multivariate analysis of OS in 85 HCC patients by Cox regression analysis

Variables	Univariate analysis			Multivariate analysis			
	**Hazard ratio**	**95% CI**	***P* value**		**Hazard ratio**	**95% CI**	***P* value**
**Gender**	1.501	0.597–3.771	0.388				
**Age, y**	0.886	0.516–1.521	0.659				
**BCLC stage**	1.355	0.769–2.390	0.293				
**Liver cirrhosis**	0.903	0.507–1.611	0.731				
**Tumor number**	1.181	0.425–3.279	0.750				
**Edmondson grade**	2.244	1.173–4.293	0.015*		1.541	0.793–2.995	0.202
**Tumor size**	2.153	1.180–3.929	0.012*		1.805	0.982–3.320	0.057
**lncRNA UBE2CP3**	3.235	1.620–6.459	0.001*		2.718	1.333–5.540	0.006*

Abbreviations : OS, overall survival; BCLC, Barcelona Clinic Liver Cancer; CI, confidence interval; *P values less than 0.05 were considered statistically significant.

### LncRNA UBE2CP3 promotes the invasion and migration of HCC cell *in vitro*

**Figure 2:**
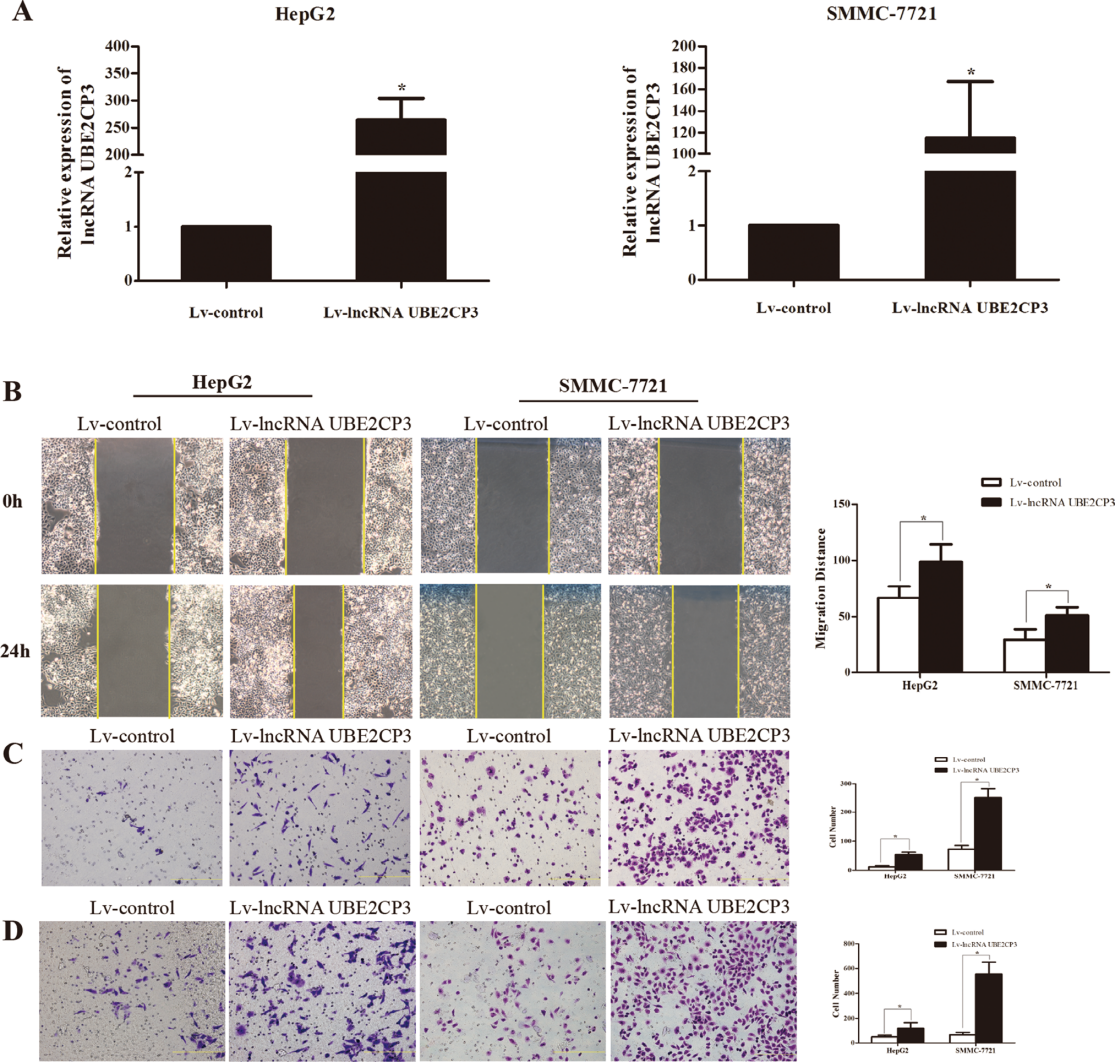
Stable over-expression of lncRNA UBE2CP3 promotes HCC cell migration and invasion *in vitro* (**A**) HepG2 and SMMC-7721 cells were infected with lentivirus carrying the lncRNA UBE2CP3 gene. HepG2 and SMMC-7721 cells stably over-expressing lncRNA UBE2CP3 were screened by qRT-PCR. (**B**) After over-expression of lncRNA UBE2CP3 in HepG2 and SMMC-7721 cells, the cell migration was assessed by wound healing assays. (**C**) *Trans-well* migration assay was performed in lncRNA UBE2CP3-over-expressing HepG2 and SMMC-7721 cells. (**D**) *Trans-well* invasion assay was performed in lncRNA UBE2CP3-over-expressing HepG2 and SMMC-7721 cells. Data are presented as mean ± SD for at least three independent experiments. *P < 0.05.

**Figure 3:**
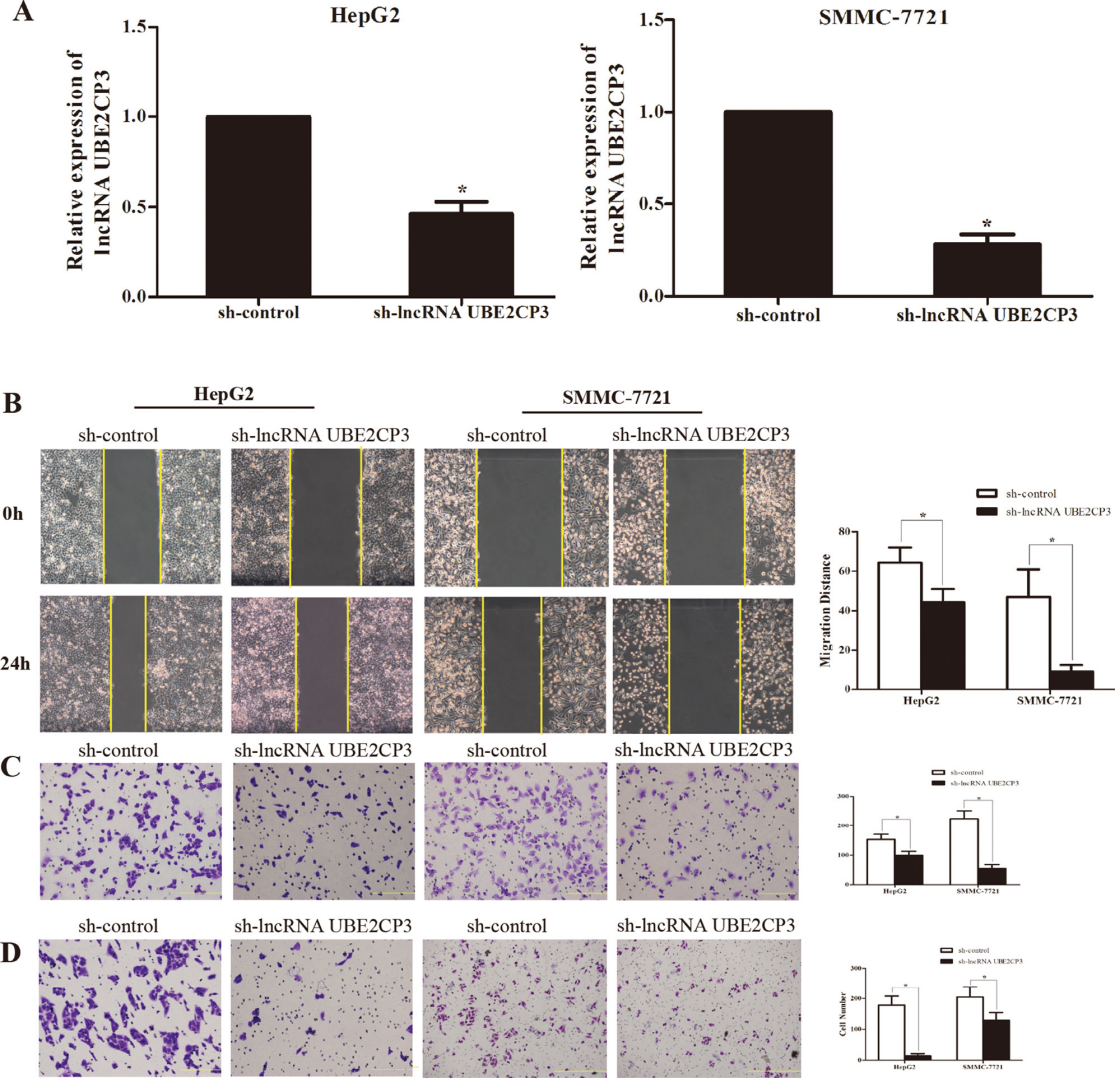
Stable suppression of lncRNA UBE2CP3 inhibits the migration and invasion of HCC cells *in vitro* (**A**) LncRNA UBE2CP3 expression was detected in HepG2 and SMMC-7721 cells by qRT-PCR after transfected with lentivirus encoding lncRNA UBE2CP3 short hairpin or a scrambled shRNA. (**B**) After knockdown of lncRNA UBE2CP3 in HepG2 and SMMC-7721 cells, cell migration was assessed by wound healing assays. (**C**) *Trans-well* migration assay was performed in lncRNA UBE2CP3-suppressed HepG2 and SMMC-7721 cells. (**D**) *Trans-well* invasion assay was performed in lncRNA UBE2CP3-suppressed HepG2 and SMMC-7721 cells. Data are presented as mean ± SD for at least three independent experiments, *P < 0.05.

The enhanced expression of lncRNA UBE2CP3 in HCC tissue implied that lncRNA UBE2CP3 may play an important role in HCC progression. The effects of lncRNA UBE2CP3 on cell invasion and migration were examined by gain- and loss-of-function studies in HCC cells. We developed HepG2 and SMMC-7721 cell lines with stably over-expressed and silenced lncRNA UBE2CP3 (Figure 2A and Figure 3A). To gain an insight into the role of lncRNA UBE2CP3 in cell invasion and migration, *trans-well* experiments and wound healing assays were performed. We observed that up-regulation of lncRNA UBE2CP3 significantly enhanced the invasion and migration potential of both HepG2 and SMMC-7721 cells compared with control group (Figure 2B–2D). In contrast, reduced expression of lncRNA UBE2CP3 dramatically decreased the invasive and migratory abilities of HCC cells (Figure 3B–3D). In addition, we investigated the effect of lncRNA UBE2CP3 on cell proliferation and cell apoptosis and observed no significant difference (Supplementary Figure 1). Thus, these results demonstrate that lncRNA UBE2CP3 may promote HCC cell invasion and migration *in vitro.*

### LncRNA UBE2CP3 induces the epithelial to mesenchymal transition (EMT) in HCC

**Figure 4:**
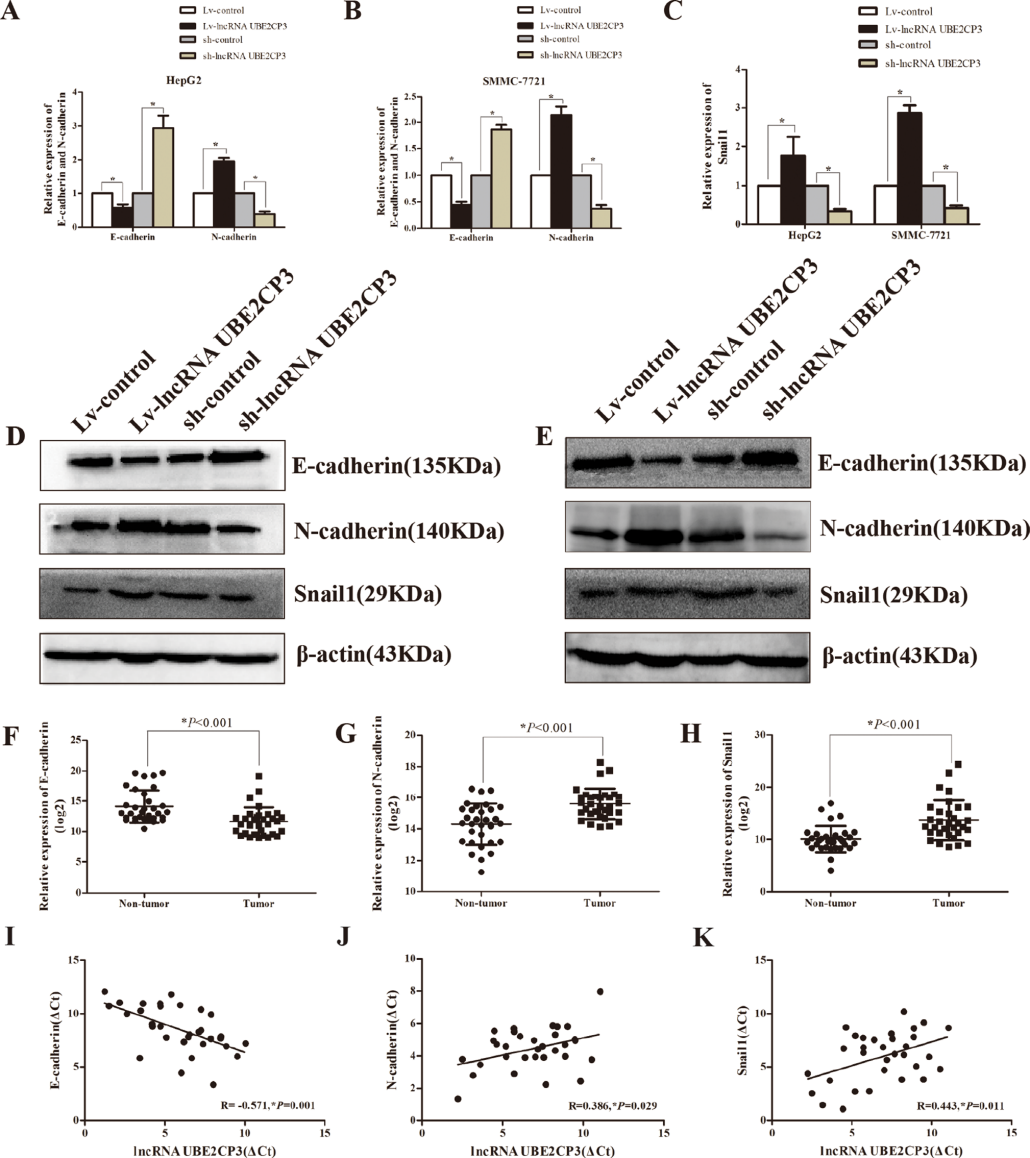
LncRNA UBE2CP3 induces the process of EMT in HCC (**A**) The relative expression levels of E-cadherin and N-cadherin were detected in HepG2 cells over-expressing or knockdown lncRNA UBE2CP3 and control cells by qRT-PCR. (**B**) The relative expression levels of E-cadherin and N-cadherin were detected in SMMC-7721cells over-expressing or knockdown lncRNA UBE2CP3 and control cells by qRT-PCR. (**C**) The relative expression levels of Snail1 were detected in HepG2 and SMMC-7721cells over-expressing or knockdown lncRNA UBE2CP3 and control cells by qRT-PCR. (**D**) The expression of E-cadherin, N-cadherin and Snail1 were examined by western blot analysis in HepG2 cells over-expressing or knockdown lncRNA UBE2CP3 and control cells. (**E**) The expression of E-cadherin, N-cadherin and Snail1 were examined by western blot analysis in SMMC-7721 cells over-expressing or knockdown lncRNA UBE2CP3 and control cells. (**F–H**) The expression of E-cadherin, N-cadherin and Snail1 were detected in 32 paired HCC tissue and non-cancerous liver tissue. (**I–K**) The correlation between lncRNA UBE2CP3 transcript level and mRNA expression of E-cadherin, N-cadherin, Snail1 in 32 HCC tissue. The ΔCt values were subjected to Pearson correlation analysis. The experiments were performed in triplicate, the data are expressed as the mean ± SD.*P < 0.05.

To gain an insight into the mechanisms by which lncRNA UBE2CP3 enhances HCC cell invasion and migration, we analyzed mRNA and protein levels of EMT-related genes in HepG2 and SMMC-7721 cells following lncRNA UBE2CP3 over-expression or silencing. Increased lncRNA UBE2CP3 in HepG2 and SMMC-7721 cells decreased the expression of epithelial protein marker E-cadherin, but elevated the expression of mesenchymal markers N-cadherin (Figure 4A and 4B). In contrast, knockdown of lncRNA UBE2CP3 induced the expression of E-cadherin but suppressed N-cadherin expression (Figure 4A and 4B). As Snail1 is reported to be a transcription factor of E-cadherin, we investigated the relationship between Snail1 and lncRNA UBE2CP3. The results showed that lncRNA UBE2CP3 was positively correlated with the expression of Snail1 (Figure 4C). All the above results were further confirmed at the protein level both in HepG2 and SMMC-7721 cells (Figure 4D and 4E). In order to confirm the relationship between E-cardherin, N-cadherin, Snail1 and lncRNA UBE2CP3, we detected their expression in 32 HCC tissue and paired non-cancerous liver tissue from Cohort 1. Consistent with our findings, N-cadherin and Snail1 were markedly elevated in HCC tissue, whereas E-cadherin was down-regulated compared to non-cancerous liver tissue (Figure 4F–4H). Moreover, a negative correlation was observed between E-cadherin and lncRNA UBE2CP3 (R = –0.571, *P* = 0.001) whereas positive correlations were observed between N-cadherin (R = 0.386, *P* = 0.029), Snail1 (R = 0.443, *P* = 0.011) and lncRNA UBE2CP3 (Figure 4I–4K).

### LncRNA UBE2CP3 promotes the metastasis of HCC *in vivo*

**Figure 5:**
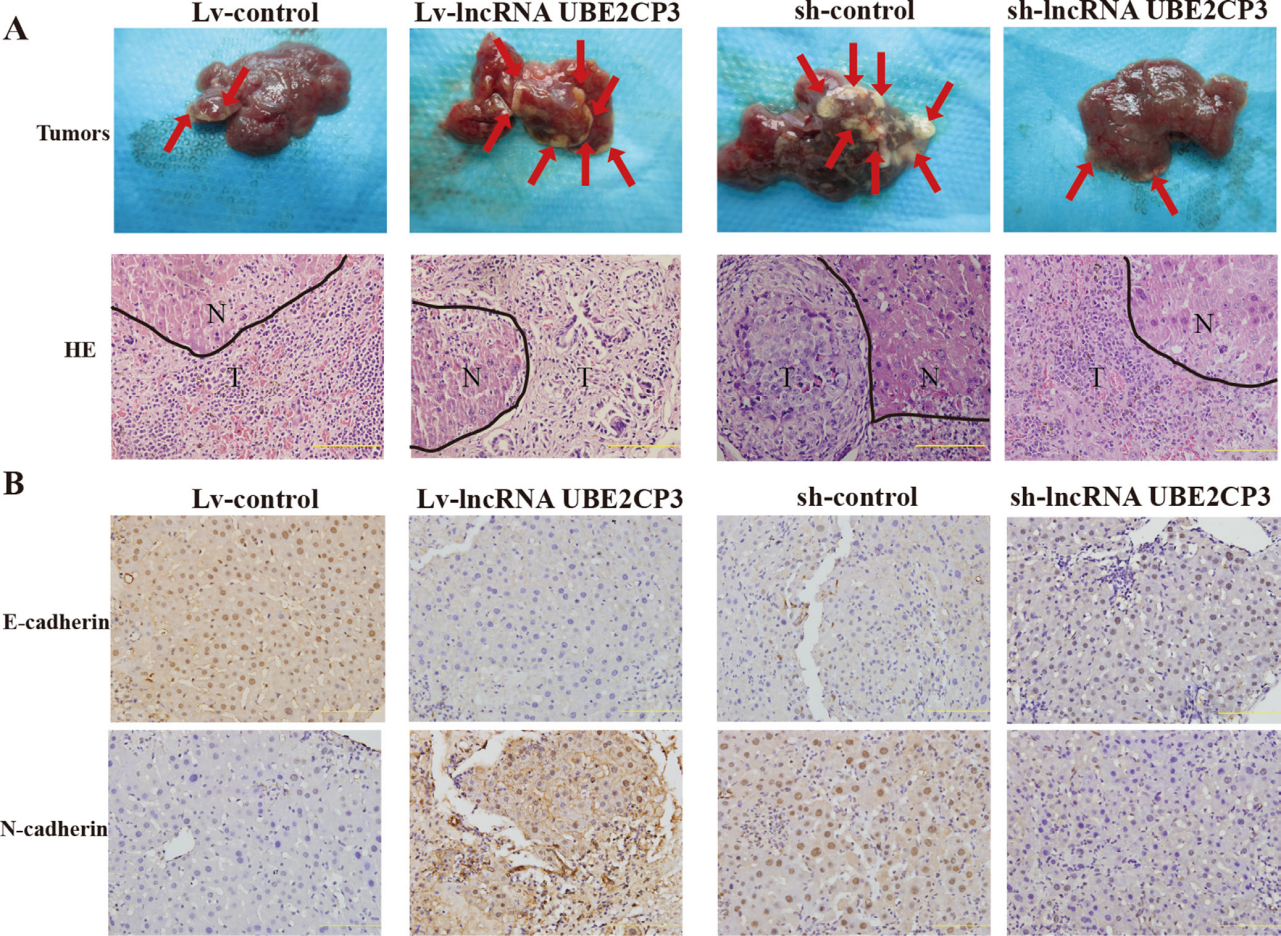
LncRNA UBE2CP3 enhances the metastasis of HCC *in vivo* (**A**) Images of intrahepatic metastasis tumors that developed in metastasis models 5 weeks after injection of lncRNA UBE2CP3-overexpressing or lncRNA UBE2CP3-suppressing or the corresponding lentivirus control HepG2 cells (upper panel). The lower panel is the HE-stained paraffin-embedded sections of the tumors (N, normal; T, tumor). (**B**) IHC analysis of E-cadherin (upper panel) and N-cadherin (lower panel) in the liver tissue from the metastasis model.

In order to determine the effect of lncRNA UBE2CP3 on metastasis *in vivo*, we injected HepG2 cells stably transfected with Lv-lncRNA UBE2CP3 (sh-lncRNA UBE2CP3) or Lv-control (sh-control) into the spleen of nude mice and intrahepatic metastasis was assessed. Mice injected with cells over-expressing lncRNA UBE2CP3 demonstrated a significant increase in intrahepatic metastasis compared to those injected with cells transfected with Lv-control (Figure 5A). Furthermore, the level of well-defined epithelial protein marker E-cadherin was markedly decreased, whereas the mesenchymal marker N-cadherin was highly detected in the liver tissue from the metastasis model (Figure 5B). Consistent with the above results, knockdown of lncRNA UBE2CP3 inhibited intrahepatic metastasis (Figure 5A). Conversely, E-cadherin was up-regulated, whereas N-cadherin was reduced in the silenced lncRNA UBE2CP3 group compared to the sh-control (Figure 5B). Xenograft models in nude mice with cells over-expressing and silencing lncRNA UBE2CP3 demonstrated that lncRNA UBE2CP3 was not involved in HCC tumor growth (Supplementary Figure 2). These findings confirmed that lncRNA UBE2CP3 may induce EMT and increase cancer metastasis in HCC.

### Serum concentration of lncRNA UBE2CP3 is increased in HCC patients

**Figure 6:**
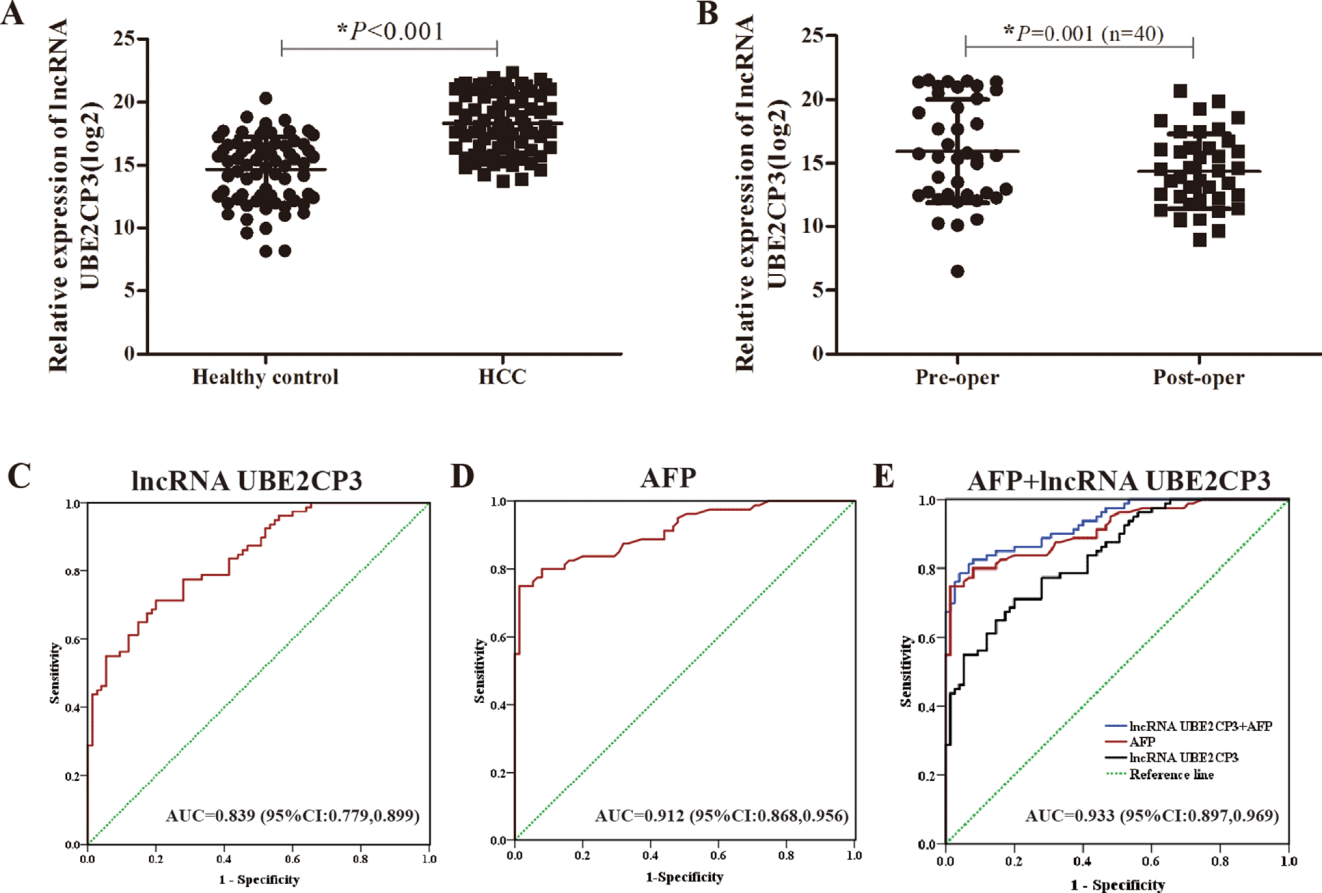
Detection of serum lncRNA UBE2CP3 in patients with HCC (**A**) Relative expression of circulating lncRNA UBE2CP3 between healthy control group (*n* = 75) and HCC group (*n* = 80). (**B**) Relative expression of serum lncRNA UBE2CP3 in 40 pairs preoperation and postoperation HCC patients. (**C**) ROC curves for serum lncRNA UBE2CP3 (AUC = 0.839; 95%Cl: 0.779,0.899; *P* < 0.001). (**D**) ROC curves for AFP (AUC = 0.912; 95%Cl: 0.868,0.956; *P* < 0.001). (**E**) ROC curves for combination of lncRNA UBE2CP3 and AFP (AUC = 0.933; 95%Cl: 0.897,0.969; *P* < 0.001).

In a pilot experiment, blood samples obtained from healthy volunteers (*n* = 75), and HCC patients (*n* = 80) were tested by qRT-PCR to check for the presence of serum lncRNA UBE2CP3. The lncRNA UBE2CP3 concentration was markedly increased in HCC patients compared with healthy volunteers (Figure 6A). Additionally, we compared the preoperative and postoperative concentrations of lncRNA UBE2CP3 in 40 HCC patients. The postoperative concentration of lncRNA UBE2CP3 was significantly lower (Figure 6B). To estimate the feasibility of serum lncRNA UBE2CP3 as a diagnostic tool for HCC, we performed receiver operating characteristic (ROC) curves. The result showed that the area under the ROC curves (AUC) was 0.839 in this model (Figure 6C). Since alpha fetoprotein (AFP) is still the most widely used biomarker for diagnosing HCC at present, we performed ROC curves for AFP and the AUC was 0.912 (Figure 6D). In addition, we determined whether the combination of lncRNA UBE2CP3 and AFP could provide a more effective diagnosis for HCC. The result indicated that a combination of lncRNA UBE2CP3 and AFP yielded an AUC of 0.933 (95% CI: 0.897–0.969, *P* < 0.001), which was improved as compared to lncRNA UBE2CP3 or AFP alone (Figure 6E).

## DISCUSSION

HCC is one of the most common cancers worldwide with a poor prognosis. The clinical symptoms are not commonly observed in the early stage of HCC development in most cases. Thus, the exploration of early biomarkers and therapeutic targets for HCC is of great importance. Many studies have shown that lncRNAs participate in cancer progression, including HCC.

In the present study, it was confirmed that the expression of lncRNA UBE2CP3 was enhanced in HCC tissue compared to their adjacent non-cancerous tissue. Clinical data demonstrated that lncRNA UBE2CP3 expression was positively correlated with Edmondson grade and potentially correlated with HCC metastasis. HCC patients with high levels of lncRNA UBE2CP3 have worse prognosis than those with low levels of lncRNA UBE2CP3. Furthermore, multivariate analysis results showed that lncRNA UBE2CP3 expression was an independent prognostic factor for HCC patients. We explored the biological function of lncRNA UBE2CP3 by gain- and loss-of-function studies. It was uncovered that enforced expression of lncRNA UBE2CP3 in HCC cells stimulated invasion and migration of HCC *in vitro* and *in vivo*. Therefore, our results demonstrate that lncRNA UBE2CP3 may serve as an oncogene in HCC.

It has been well established that HCC is one of the most common cancers worldwide and is associated with high prevalence and lethality, which is caused by several factors such as HBV infections, alcoholic abuse and aflatoxin exposure. Tumor size, tumor relapse, BCLC stage, and AFP were considered to be important predictors of tumor recurrence. Our findings suggest that high lncRNA UBE2CP3 expression levels are not only significantly correlated with Edmondson grade but also correlated with smoking and alcohol. Similarly, Wang et al.[19] reported that the expression of lncRNA AOCP4 was correlated with smoking in HCC patients. Alcohol abuse had been considered as the most common cause of liver cirrhosis, which was associated with an increased risk for HCC[20]. Moreover, Kaplan-Meier and log-rank test analysis suggested that high lncRNA UBE2CP3 expression levels predicted a poor prognosis. Multivariate analysis results suggested that lncRNA UBE2CP3 may be an independent prognostic factor for OS in HCC patients. These data strongly suggest that lncRNA UBE2CP3 contributes to HCC malignant progression and may serve as a predictor for HCC prognosis.

A number of studies have been conducted on the mechanisms underlying carcinogenesis and disease progression, including lncRNAs [21, 22]. To date, some characterized human lncRNAs have been linked to a spectrum of biological functions and the disruption of these functions may play a crucial role in the progression of HCC. Wang et al. [23] reported that lncRNA-hPVT1 is up-regulated in HCC tissue and that its high expression level is related to cell proliferation. Furthermore, Cao et al. [24] identified differentially expressed lncRNAs in HCC samples and adjacent non-tumor tissue from seven patients and found that lincRNA-UFC1 acted as an oncogene to promote cell growth and inhibit apoptosis. Our previous study showed that HBX-related lncRNA DBH-AS1 promotes cell proliferation and cell survival through activating the MAPK signaling pathway in HCC [25].In the present study, we analyzed three pairs of human HCC and adjacent non-cancerous tissue with a microarray. Consistent with the microarray data of Cao et al. [24] and two human lncRNA microarray datasets (GSE55191 and GSE58043) [26], we found that the lncRNA UBE2CP3 was up-regulated in HCC tissue. *In vitro* and *in vivo* assays showed that over-expression of lncRNA UBE2CP3 promoted cell invasion and migration, whereas it had no impact on cell cycle or cell apoptosis. Increasing evidence suggests that there are multiple factors, including aberrant key molecules and signaling pathways, that affect HCC cell cycle and cell apoptosis [27, 28]. Our results suggest that there must be additional factors, aside from lncRNA UBE2CP3, involved in HCC cell cycle and apoptosis.

EMT is a complex process that involves loss of epithelial cells polarity and cell-to-cell contact, and the acquisition of more migratory and invasive properties to become mesenchymal cells [29]. EMT plays an important role in cancer progression, metastasis, and drug resistance [30]. E-cadherin is the marker of epithelial cells whereas N-cadherin is a well-defined mesenchymal cells marker. Given the results we obtained in the functions of lncRNA UBE2CP3 relating to cell invasion and migration, we tested the expression of E-cadherin, N-cadherin in mRNA and protein levels. Increased lncRNA UBE2CP3 decreased the expression of E-cadherin, but elevated the expression of N-cadherin. By contrast, knockdown of lncRNA UBE2CP3 showed the opposite results. In agreement with these results, the expression level of E-cadherin was markedly decreased, whereas N-cadherin was highly detected by IHC analysis of the liver tissue from the metastasis model. As Snail1 is the transcription factor of E-cadherin [31, 32], we tested the relationship between Snail1 and lncRNA UBE2CP3. As expected, over-expression of lncRNA UBE2CP3 increased the expression of Snail1 both at the mRNA and protein level. Moreover, our tissue results were consistent with this finding. Altogether, these data indicate that lncRNA UBE2CP3 is involved in the process of EMT, with further studies required to identify the precise mechanism regulating this process.

Numerous studies have elucidated that the localization of lncRNAs in the cytoplasm plays a pivotal role in post-transcriptional regulation of genes functioning as partners with miRNAs, mRNAs or proteins that regulate target genes involved in the development of HCC [33, 34]. In our study, the ISH results revealed that lncRNA UBE2CP3 was located mainly in cytoplasm. Thus, we speculated that cytoplasmic lncRNA UBE2CP3 may act as a sponge for miRNAs or integrate with proteins or mRNAs to regulate downstream molecules. We found that lncRNA UBE2CP3 had a binding site to miR-138-5p (data not shown). Recently, interactions between lncRNAs and miRNAs have been reported. For example, HOTTIP was reported to be negatively regulated by miR-125b to promote HCC cell proliferation and tumorigenesis [35]. Hu et al. [36] reported that lncRNA GAS5 regulated migration and invasion of HCC cells through regulating miR-21 and its targets. Additionally, miR-138-5p has been proven to be an important regulatory molecule in the process of EMT [37, 38]. Liang et al. [39] demonstrated that lncRNA H19 modulated the expression of multiple genes involved in EMT by acting as a competing endogenous RNA for miR-138 and miR-200a. Interestingly, miR-138-5p was found to be associated with metastasis of HCC [40, 41]. Thus, we hypothesized that miR-138-5p may target lncRNA UBE2CP3 to regulate the process of EMT. More detailed mechanisms should be experimentally investigated in the future.

Biomarkers for HCC have been extensively explored over the past decades [42]. Currently, AFP is considered the most frequently used marker for clinical HCC screening [43]. Moreover, novel cancer biomarkers have been confirmed, due to the development of high throughput microarray and secondary generation sequencing developments [44]. Indeed, Du et al. [45] reported that plasma miR-21 may be a reliable and non-invasive biomarker for colorectal cancer diagnosis by performing a comprehensive meta-analysis. Similarly, Ono et al. [46] revealed that the expression level of circulating miR-210 in plasma predicts early clinical recurrence in melanoma patients. Circulating lncRNAs also have been reported as biomarkers in predicting tumoral features. For example, lncRNA PCA3 has been identified as a useful invasive biomarker for the diagnosis of prostate cancer with a prognostic value [47]. A lncRNA panel including lncRNA-LET, PVT1, PANDAR, PTENP1, and linc00963, were identified to distinguish clear cell renal cell carcinoma from healthy controls[48]. However, few clinical studies have been performed on circulating lncRNAs in HCC. In the present study, we detected a high expression level of lncRNA UBE2CP3 in the serum of HCC patients. We speculated that lncRNA UBE2CP3 may be a new fluid biomarker for HCC. Accordingly, we found that lncRNA UBE2CP3 in the postoperative phase was significantly lower than that in the preoperative phase. We applied the ROC curves to analyze the diagnostic value of lncRNA UBE2CP3. The results showed that the individual AUC of lncRNA UBE2CP3 was 0.839. Since AFP is the most widely used biomarker for diagnosing HCC at present, we performed the ROC curves of AFP and the individual AUC of AFP was 0.912. Moreover, a combination of lncRNA UBE2CP3 and AFP yielded an AUC of 0.933, which was improved as compared to lncRNA UBE2CP3 or AFP alone. These results suggest that the combination of lncRNA UBE2CP3 and AFP is more effective for diagnosing HCC and that lncRNA UBE2CP3 is released into the circulation. However, the mechanisms by which this occurs remain unknown. Taken together, the results of this study imply that serum lncRNA UBE2CP3 may be a potential biomarker for clinical evaluation of HCC.

The key finding of this study was that high expression of lncRNA UBE2CP3 could promote HCC cell invasion and migration and thus may be a potential biomarker for HCC. However, there are still limitations in our present study. The molecular mechanisms that regulate the role of lncRNA UBE2CP3 in the EMT process remain unclear. Further investigations such as bioinformatics analysis are needed to establish which miRNAs or mRNAs directly bind to lncRNA UBE2CP3. Additionally, our sample size for measuring serum lncRNA UBE2CP3 levels was small. A larger sample size should be used in the future.

In conclusion, we revealed that lncRNA UBE2CP3 promotes the metastasis of HCC by inducing the process of EMT. Moreover, we confirmed that serum lncRNA UBE2CP3 is increased in HCC patients. Overall, our study identified the role of lncRNA UBE2CP3 in HCC progression and survival prediction in HCC patients. These results indicate that lncRNA UBE2CP3 could be a candidate diagnostic biomarker and a target for new therapies in HCC.

## MATERIALS AND METHODS

### Patients and clinical specimens

Two independent cohorts of 131 HCC patients were enrolled in this study. In Cohort 1, fresh HCC specimens and corresponding adjacent non-cancerous tissue were obtained from 46 patients who were diagnosed with HCC between October 2012 and December 2013 at Nanfang Hospital, Southern Medical University (Guangzhou, China). In Cohort 2, formalin-fixed paraffin-embedded tissue were obtained from 85 HCC patients who initially underwent hepatectomy between January 2007 and November 2009 from the same hospital. The patients of Cohort 2 patients were followed up for 5 years following surgery. OS was defined as the interval between resection and death or the last follow-up examination. Patients’ clinical information is listed in Table 1 and Table 2. All patients provided written informed consent and the research protocol was approved by the Ethics Committee of Nanfang Hospital (Guangzhou, China).

### Serum sample collection

All serum samples (80 HCC patients) were obtained from Nanfang Hospital, Southern Medical University (Guangzhou, China), from January 2016 to September 2016. Paired plasma samples (preoperative and postoperative) were obtained from 40 HCC patients, and postoperative samples collected 10 days following surgery. All HCC patients were diagnosed by histological examination. Control samples were obtained from 75 healthy volunteers without any cancer or other health problems during health check-ups at the same hospital. All serum samples were stored at −80°C until further analysis. The relevant clinical data of all the patients were available and is listed in Supplementary Table 1. Each research subject signed a prior informed consent that was approved by the Ethics Committee of Nanfang Hospital.

### Cell lines and cell culture

HepG2 and SMMC-7721 cell lines were purchased from the Cell Bank of Type Culture Collection (CBTCC, Chinese Academy of Sciences, Shanghai, China). Cells were maintained at 37°C in a humidified incubator containing 5% CO_2_ in Dulbecco’s Modified Eagle Medium (DMEM, Gibco, Gaithersburg, MD, USA) supplemented with 10% fetal bovine serum (Gibco).

### Microarrays and computational analysis

Sample preparation and microarray hybridization were performed by Kangchen Bio-tech, (Shanghai P.R. China). Three paired fresh HCC tissue and corresponding non-cancerous tissue were used for microarray analysis. Briefly, total RNA was extracted with TRIzol® Reagent (Invitrogen, Carlsbad, CA, USA) and purified using the RNasey Mini Kit (Qiagen, Valencia, CA, USA). cDNA was synthesized and labeled before it was purified and hybridized to the microarray (Arraystar, Rockville, MD, USA). Slides were scanned with the Agilent DNA Microarray Scanner after washing and Agilent Feature Extraction software used to extract the data. Further data analysis was performed using Agilent GeneSpring GX v12.0 software. We performed a Volcano Plot filtering (Fold Change > = 2.0, *P-value* < = 0.05) between HCC and the matched non-tumor samples to identify significantly different expressions of lncRNAs. Hierarchical clustering was performed based on the differential expressions of lncRNAs using Cluster_Treeview software from Stanford University.

### RNA isolation and qRT-PCR

RNA was extracted from cultured cells or human tissue using TRIzol Reagent (Takara, Dalian, China). Serum total RNA in plasma was extracted using TRIzol LS Reagent (Magen, Guangzhou, China) according to the manufacturer’s instructions. For lncRNA UBE2CP3, first-strand cDNA was synthesized using the M-MLV Reverse Transcriptase (Promega, Madison WI, USA). For mRNAs, cDNA was generated by using the PrimeScript RT reagent kit (Takara). The RNA expression levels were measured by qRT-PCR using SYBR Green PCR Master Mix (Takara), which was performed on the ABI 7500 Fast Real Time PCR system (Applied Biosystems, Foster City, CA, USA). U6 and β-actin were used as internal controls. All results were expressed as the means ± SD of at least three independent experiments. Comparative quantification was determined using the 2^−ΔΔCt^ method. The primers used are presented in Supplementary Table 2.

### Western blot analysis

Total proteins were prepared from the samples by complete cell lysis (Keygen Biotech, Jiangsu, China) with protease and phosphatase inhibitors. Quantified protein lysates were separated on sodium dodecyl sulfate-polyacrylamide gel and transferred onto polyvinylidene difluoride membranes, blocked with 5% BSA for 1 h at room temperature, and incubated with primary antibodies overnight at 4°C. After incubation with the goat anti-rabbit secondary antibodies, the proteins were visualized using a chemiluminescence method (ECL Plus Western Blotting Detection System; Amersham Biosciences, Foster City, CA, USA). The primary antibodies are listed in Supplementary Table 3. Each experiment was performed in triplicate.

### Lentiviral construction and cell transfections

To generate cell lines stably over-expressing lncRNA UBE2CP3, HepG2 and SMMC-7721 cells were infected with the Lv-lncRNA UBE2CP3 and Lv-control viruses (LAND, Guangzhou, China). To observe the knockdown effects of lncRNA UBE2CP3 *in vitro*, HepG2 and SMMC-7721 were transfected with the shRNA-lncRNA UBE2CP3 (sh-lncRNA UBE2CP3) or control viruses (sh-control) from Obio Technology (Shanghai) Corp, Ltd. Stably infected cell lines were selected for 2 weeks using puromycin and the expression level of lncRNA UBE2CP3 was confirmed by qRT-PCR. The target sequences were described in Supplementary Table 2.

### CCK-8 assays, cell cycle analysis, and *in vivo* tumor growth assays

CCK-8 assays, cell cycle analysis, and *in vivo* tumor growth assays were performed according the previously described method [25].

### Wound healing assay

Wound healing assay was used to determine cell migration. Cells in each group were collected and resuspended in DMEM medium. Each well of a six-well plate was seeded with 5 × 10^5^ cells and cultured for 24 h to 100% confluence. Wounds were made using a 100 μl plastic pipette tip. The wound widths were measured and recorded after 0 h, 24 h following wound and photographed. The percent of wound closure was calculated for five randomly chosen fields.

### Cell invasion and migration assays

To observe the effects of lncRNA UBE2CP3 on cell migration and invasion, *trans-well* experiments were performed by using an 8-μm pore *trans-well* chamber (BD Biosciences, MA, USA) with/without Matrigel (BD Biosciences). In the invasion assay, a *trans-well* chamber was placed into a 24-well plate and coated with Matrigel. In both *trans-well* assays, cells were suspended in serum-free medium and were allowed to migrate toward the complete media supplemented with 10% fetal bovine serum after over-expression or knockdown of lncRNA UBE2CP3. After 24 h incubation at 37°C, migrated cells were fixed with 100% methanol for 30 min. Non-migrated cells were removed by cotton swabs. Next, the cells on the bottom surface of the membrane were stained with 0.1% crystal violet for 20 min. Cell numbers in 5 random fields in each replicate were counted using microscope (NiKon ECLPSE 80i system; magnification: × 200). Each assay was performed at least three times.

### Metastasis model *in vivo*

To observe the effects of lncRNA UBE2CP3 on metastasis *in vivo*, we constructed stable cell lines with lncRNA UBE2CP3 expression by lentiviral construction. Cells were collected by trypsinization, washed twice with PBS, and resuspended with serum-free medium. A total of 1 × 10^7^ HepG2 cells stably transfected with Lv-lncRNA UBE2CP3 (sh-lncRNA UBE2CP3) or Lv-control (sh-control) in 0.1 mL of DMEM medium, injected into the spleen of nude mice and intrahepatic metastasis was assayed for each group. Male BALB/C nude mice (4–6 weeks) were purchased from Central Laboratory of Animal Science, Southern Medical University (Guangzhou, China) and raised in a specific pathogen-free facility. All *in vivo* experiments were performed according to the guidelines for the use of laboratory animals and approved by the Institutional Animal Care and Use Committee of Nanfang Hospital.

### Immunohistochemistry

IHC for E-cadherin and N-cadherin were performed on paraffin sections using a primary antibody against E-cadherin and N-cadherin (Cell Signaling Technology) and a horseradish peroxidase-conjugated rabbit anti-goat antibody (Maixin, Fuzhou, China), and the proteins *in situ* visualized with 3, 3-diaminobenzidine and analyzed using a bright field microscope.

### *In situ* Hybridization

The ISH probe used for detecting lncRNA UBE2CP3-labeled digoxin was designed and synthesized by Exiqon (Shanghai, China). The probe sequence was designed as TGTGTCACTAGGCATTGT. ISH was performed using the ISH Kit (Boster Bio-Engineering Company, Wuhan, China) according to the manufacturer’s instructions. The ISH staining regions for lncRNA UBE2CP3 were scored by two pathologists, blinded to the clinical parameters. The score standard for the staining intensity was defined as follows: 0 (negative), 1 (weak), 2 (medium), 3 (strong). The score of staining extent was 0 (10%), 1 (11%–25%), 2 (26%–50%), 3 (51%–75%), and 4 (76%–100%). The final lncRNA UBE2CP3 expression score was calculated with the intensity score and extent score, ranging from 0 to 7. The high expression group (positive group) was defined as a total score of 3 or higher [24].

### Statistical analysis

Statistical analysis was performed using SPSS 13.0 software (SPSS Inc., Chicago, IL, USA) and GraphPad software (GraphPad Software, Inc., La Jolla, CA, USA). Survival curves were calculated via Kaplan-Meier and log-rank tests. Each variable with statistical significance in the univaviate analysis was entered into multivariate models for estimating their independent prognostic values on cancer. A chi-square test was performed to analyze the relationship between lncRNA UBE2CP3 expression levels and clinicopathological characteristics. Student’s *t-test* and multi-way classification ANOVA tests were performed for results from qRT-PCR experiments, wound healing, *trans-well* invasion and migration assays. Correlations between lncRNA UBE2CP3 and E-cadherin, N-cadherin, Snail1 were analyzed by Pearson correlation analysis. *P* < 0.05 was considered to be statistically significant.

## SUPPLEMENTARY MATERIALS FIGURES AND TABLES



## Abbreviations

HCC: hepatocellular carcinoma
lncRNA: long non-coding RNA
qRT-PCR: quantitative real-time polymerase chain reaction
ISH: *in situ* hybridization
EMT: epithelial to mesenchymal transition
IHC: immunohistochemistry
OS: overall survival
ROC: receiver operating characteristic
AUC: area under the ROC curves
AFP: alpha fetoprotein
