# Supplementary material for: Long non-coding RNA UBE2CP3 promotes tumor metastasis by inducing epithelial-mesenchymal transition in hepatocellular carcinoma
Source: Oncotarget. 2017 Jun 16;8(39):65370–85. doi: 10.18632/oncotarget.18524 (PMC5630337; doi:10.18632/oncotarget.18524)
Supplement: Supplementary file 1 [file oncotarget-08-65370-s001.pdf]

Long non-coding RNA UBE2CP3 promotes tumor metastasis by inducing epithelial-mesenchymal transition in hepatocellular carcinoma

Supplementary Materials

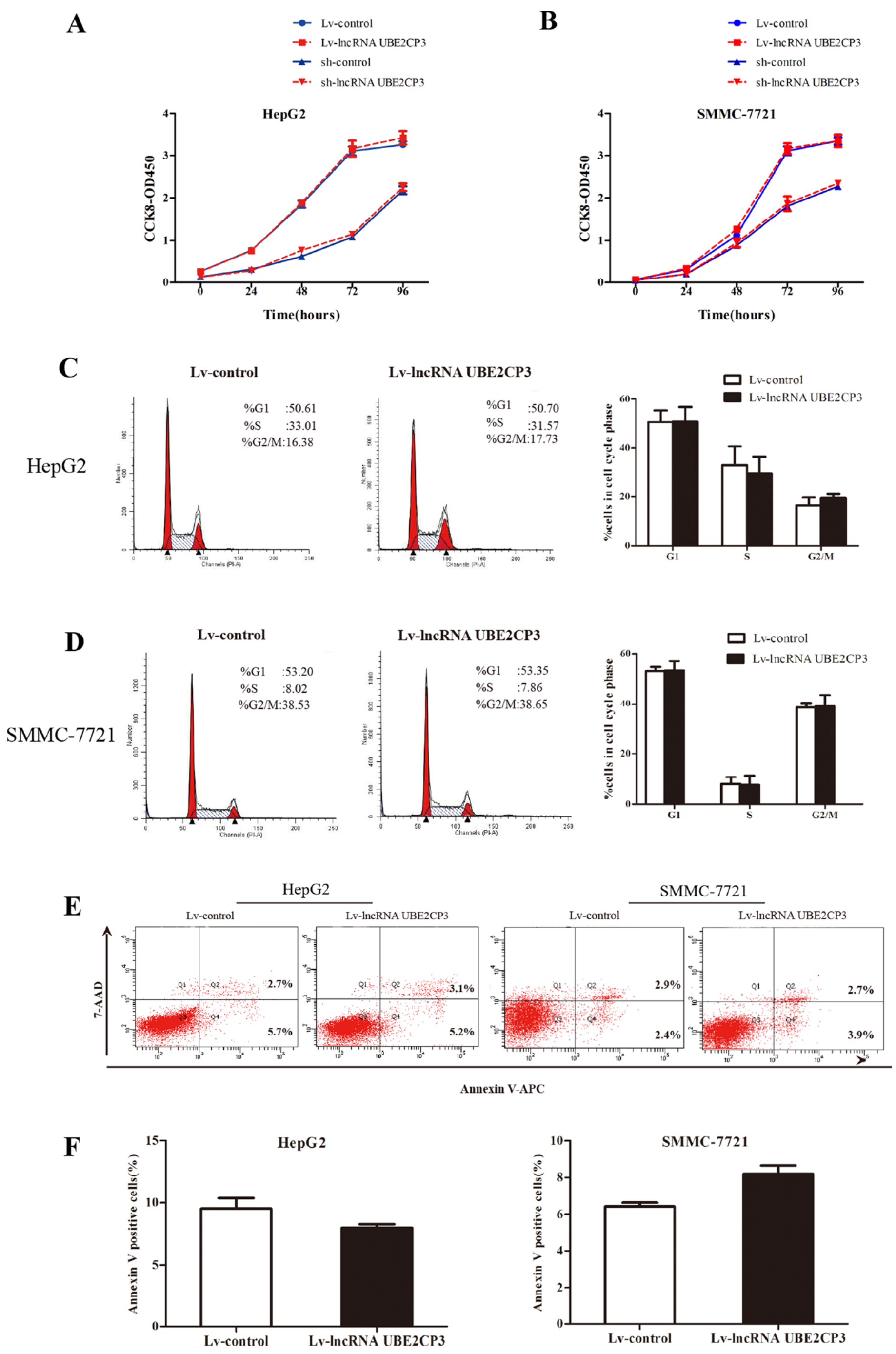

Supplementary Figure 1: LncRNA UBE2CP3 shows no effect on cell proliferation, cell cycle progression and cell apoptosis *in vitro*. (**A–B**) After over-expression and knockdown of lncRNA UBE2CP3 in HepG2 and SMMC-7721 cells, the cell viability was assessed by CCK-8 assays daily for 4 days. (**C–D**) Cell cycle analysis of over-expression of lncRNA UBE2CP3 in HepG2 and SMMC-7721 cells. (**E–F**) Cell apoptosis analysis of over-expression of lncRNA UBE2CP3 in HepG2 and SMMC-7721 cells.

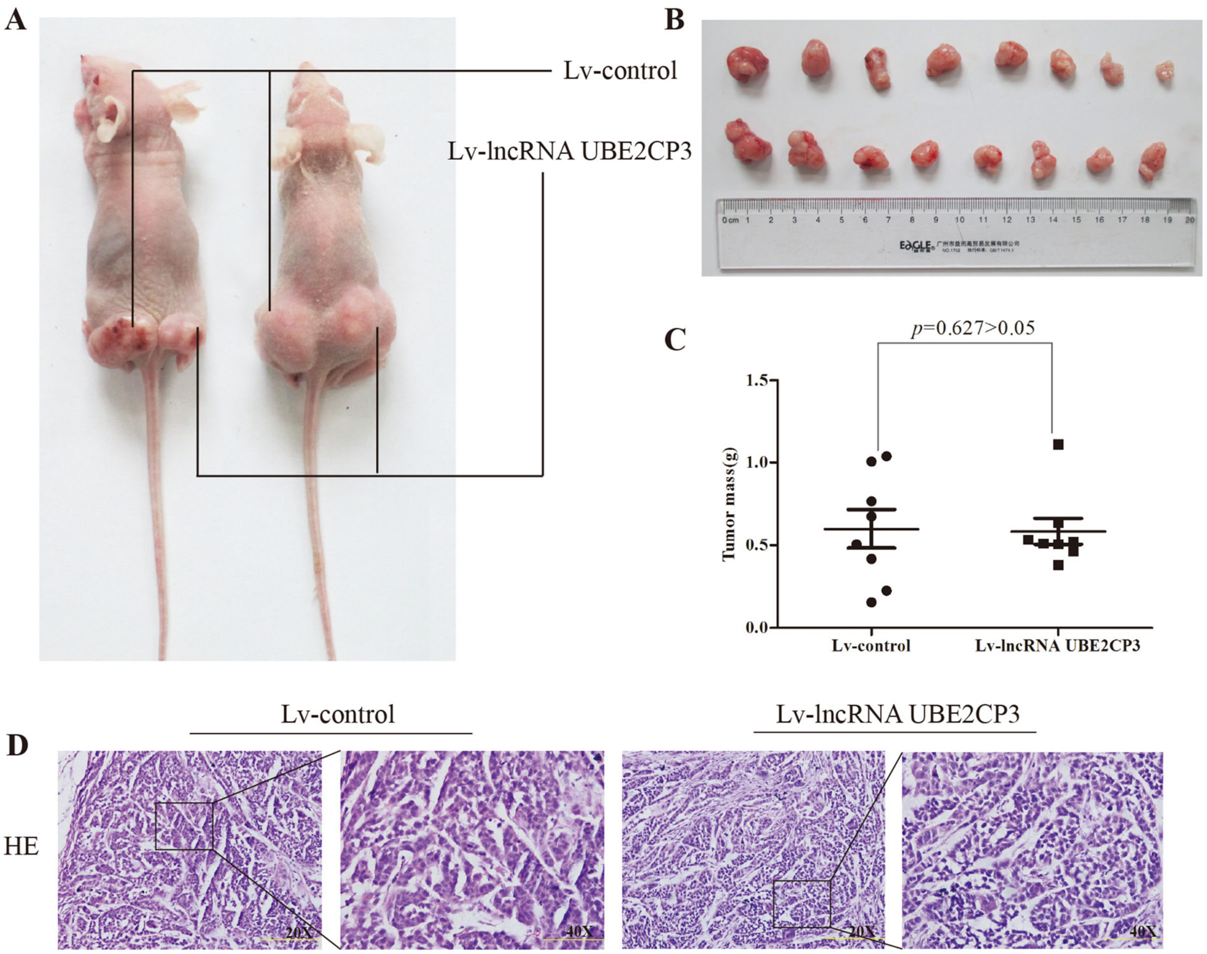

Supplementary Figure 2: LncRNA UBE2CP3 has no effect on tumor growth *in vivo*. (**A–B**) Photographs of tumors and images that developed in xenograft-transplanted nude mouse tumor models 5 weeks after injection of lncRNA UBE2CP3-over-expressing or control HepG2 cells. (**C**) Weights of xenografts established by subcutaneous transplantation with Lv-lncRNA UBE2CP3-overexpressing and Lv-control HepG2 cells 5 weeks after cell injection. (**D**) HE-stained paraffin-embedded sections obtained from xenografts.

Supplementary Table 1: Clinicopathologic characteristics of 80 serum HCC patients

Feature	*N*(%)
**Age,y**	
**< = 55**	48（60.0）
**> 55**	32（40.0）
**Gender**	
**Male**	71（88.7）
**Female**	9（11.3）
**HBsAg**	
**Positive**	69（86.2）
**Negative**	11（13.8）
**Tumor size,cm**	
**< = 5**	39（48.7）
**> 5**	41（51.3）
**AFP level, ng/ml**	
**< = 20**	56（70.0）
**> 20**	24（30.0）
**Cirrhosis**	
**Yes**	61（76.2）
**No**	19（23.8）
**Tumors, n**	
**Solitary**	70（87.5）
**Multiple**	10（12.5）
**Smoking**	
**Yes**	33（41.2）
**No**	47（58.8）
**Alcohol**	
**Yes**	24（30.0）
**No**	56（70.0）

Supplementary Table 2: Sequences of primers and shRNA used in this study

Name		Sequences
**qRT-PCR primers**		
**lncRNA UBE2CP3**	sense	AAGTGGTCTGCCCTGTATGATG
	antisense	GAGCTATCAATGTTGGGTTTGC
**E-cadherin**	sense	GTCCTGGGCAGACTGAATTT
	antisense	GACCAAGAAATGGATCTGTGG
**N-cadherin**	sense	TGGACCATCACTCGGCTTA
	antisense	ACACTGGCAAACCTTCACG
**Snail1**	sense	ACCACTATGCCGCGCTCTT
	antisense	GGTCGTAGGGCTGCTGGAA
**β-actin**	sense	TGGCACCCAGCACAATGAA
	antisense	CTAAGTCATAGTCCGCCTAGAAGCA
**U6**	sense	CTCGCTTCGGCAGCACA
	antisense	AACGCTTCACGAATTTGCGT
**shRNA sequences**<br>**sh-lncRNA UBE2CP3**	CCGGCCCAGGGTAATATCTGCCTCTCAAGAGAAGGCAGATATTACCCTGGGTTTTTG	

Supplementary Table 3: Information of antibodies used in this study

Antibody	WB	IHC	Specificity	Company
**E-cadherin (24E10)**	1:1000	1:400	Rabbit monoclonal	Cell Signaling Technology
**N-cadherin (D4R1H)**	1:1000	1:125	Rabbit monoclonal	Cell Signaling Technology
**Snail1 (WL01863)**	1:1000	/	Rabbit polyclonal	Wanleibio
**β-actin (bs-0061R)**	1:2000	/	Rabbit polyclonal	Bioss

WB, western blot; IHC, immunohistochemistry.
